# Artificial Intelligence Applications in Pneumonia: Diagnosis and Outcome Prediction

**DOI:** 10.1007/s13665-026-00411-9

**Published:** 2026-05-09

**Authors:** Mengou Zhu, Melissa J. Bak, Catherine A. Gao

**Affiliations:** 1 Department of Medicine, Northwestern University Feinberg School of Medicine, Chicago, IL USA; 2 Division of Pulmonary and Critical Care, Department of Medicine, Northwestern University Feinberg School of Medicine, Chicago, IL USA

**Keywords:** Artificial intelligence, Pneumonia diagnosis, Machine learning, Deep learning, Outcome prediction, Neural networks, Electronic health records

## Abstract

**Purpose of Review:**

This review explores the current use of artificial intelligence (AI) in pneumonia diagnosis and outcome prediction. It aims to highlight advancements in AI technologies, focusing on both imaging and electronic health record-based approaches, their impact on improving diagnostic accuracy, and predicting clinical outcomes.

**Recent Findings:**

AI systems can both diagnose pneumonia and predict disease severity, mortality, and other key outcomes, such as hospital length of stay and readmission risk. These tools integrate diverse data sources, including demographics, lab markers, and vital signs, to enhance clinical decision-making. Recent imaging models using neural networks demonstrated high accuracy in detecting pneumonia from chest X-rays and CT scans, surpassing human radiologists in some cases. However, challenges remain, including inconsistencies in pneumonia labeling, data quality issues, and the limited generalizability of models across different healthcare settings.

**Summary:**

AI holds significant potential to improve pneumonia diagnosis and patient outcomes, though challenges such as data biases, model interpretability, and standardization remain. Continued research is needed to address these limitations and optimize AI integration into clinical practice.

## Introduction

Pneumonia, acute respiratory infection of the lower airways, continues to be a significant global health burden [1]. According to the World Health Organization (WHO), pneumonia is responsible for over 2.5 million deaths annually, with the highest burden in low- and middle-income countries [2]. The global incidence of pneumonia is estimated at over 100 million cases per year, with significant morbidity, especially in vulnerable populations such as young children, the elderly, and individuals with compromised immune systems [3]. Timely and accurate diagnosis and outcome prediction is crucial in reducing both mortality and morbidity. However, traditional diagnostic methods often fall short in rapidly providing reliable results [4].

Conventional diagnostic approaches, such as clinical judgment and radiographic imaging, have been the cornerstone of pneumonia detection for decades. However, these methods come with notable limitations. Radiographic interpretation, which relies heavily on the expertise of radiologists, can be challenging due to variability in image quality, human error, and the complexity of distinguishing pneumonia from other pulmonary conditions, such as chronic obstructive pulmonary disease (COPD) or pulmonary edema [5, 6]. Additionally, clinical judgment alone is prone to subjectivity and may be influenced by a clinician’s experience, patient comorbidities, and presentation [7]. Moreover, diagnostic delays due to the time required for laboratory tests, particularly microbiological cultures [8], can result in suboptimal patient outcomes, particularly in severe cases.

In terms of pneumonia outcome prediction, the most widely used approaches currently are scoring systems such as the Pneumonia Severity Index (PSI) and CURB-65 [9, 10]. Some other scoring systems, such as Sequential Organ Failure Assessment (SOFA) and Acute Physiology and Chronic Health Evaluation (APACHE) III, which were initially designed for risk stratification in related clinical conditions such as sepsis and critical illness, are sometimes also used for pneumonia risk stratification and outcome prediction [11, 12]. These existing scoring systems do not account for complex interactions between clinical features or non-linear risk patterns as observed in real-world clinical data. They also tend to reflect a snapshot of a static clinical timestamp and fail to capture the dynamic change in a patient’s clinical course, which can lead to delays in recognition of poor outcome. More accurate pneumonia outcome prediction allows for accurate risk stratification, which allows for appropriate allocation of clinical resources; it also allows for early identification of possible deterioration, which allows for additional diagnostic workup and therapeutic adjustments.

Artificial intelligence (AI), particularly machine learning (ML) and deep learning (DL) models, has emerged as a promising tool to address the shortcomings of traditional diagnostic and prognostic methods [13]. Artificial intelligence (AI) is a broad term referring to computational methods that enable machines to perform tasks that typically require human intelligence, with machine learning (ML) representing a subset of AI in which algorithms learn patterns from data to make predictions or decisions (e.g., logistic regression [LR], random forests [RF], and extreme gradient boosting [XGBoost]). Deep learning (DL), a further subset of ML, uses multi-layered neural networks to model complex relationships in high-dimensional data such as medical images and clinical text (e.g., convolutional neural networks [CNN] and recurrent neural networks [RNN]). The application of AI in healthcare has seen significant growth in recent years [14]. Additionally, the ability to integrate and analyze large datasets enables a more comprehensive and personalized approach to pneumonia diagnosis and outcome prediction. These tools can assist in detecting pneumonia at an earlier stage, predict disease progression, and support clinicians in making evidence-based decisions about treatment strategies.

**Fig. 1:**
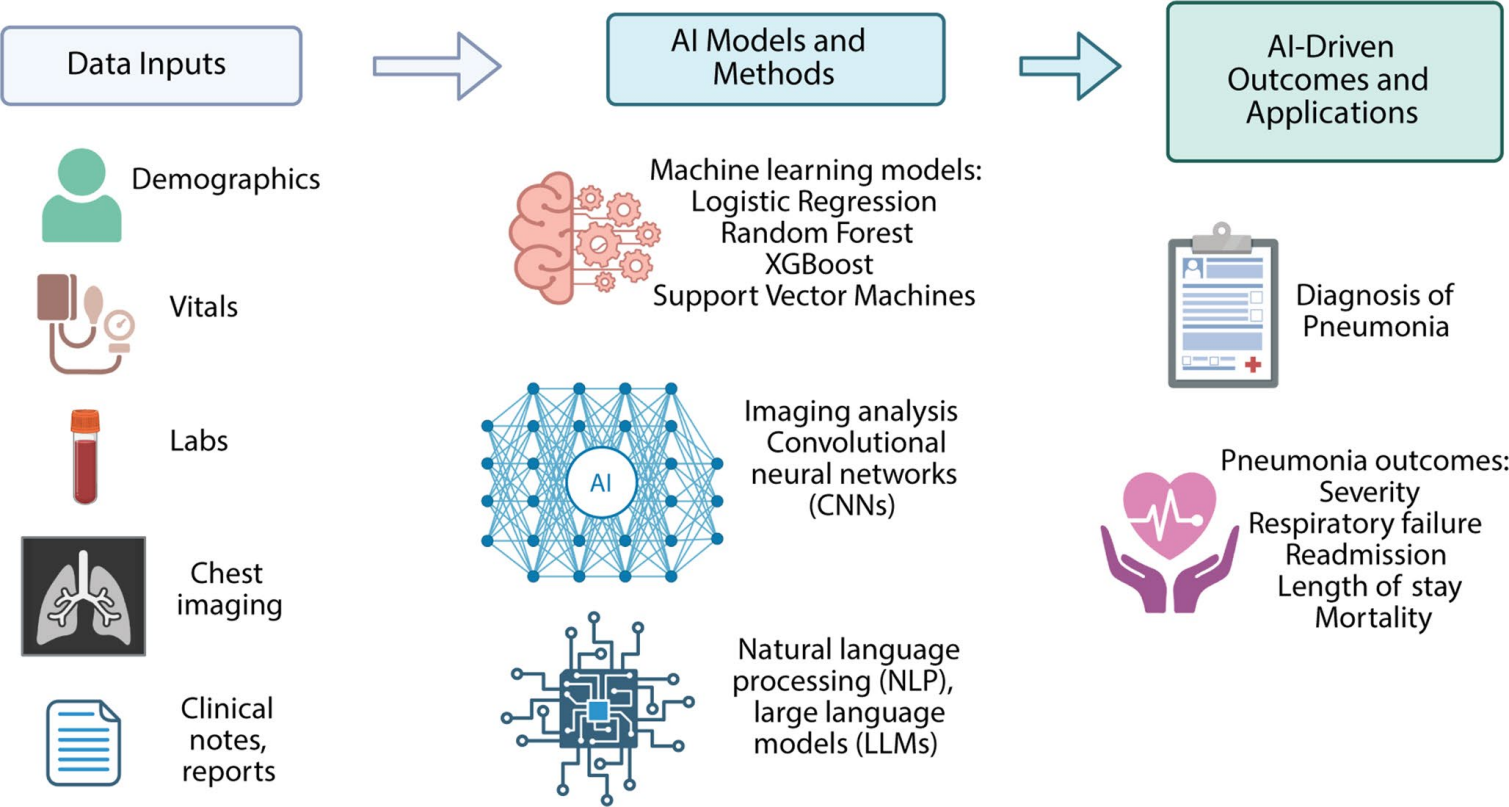
Overview of common data inputs, modeling approaches, and clinical applications of artificial intelligence (AI) in pneumonia. Input data include demographic characteristics, vital signs, laboratory results, chest imaging, and unstructured clinical text (e.g., notes and reports). These data are processed using machine learning (ML) algorithms such as logistic regression, random forests, gradient boosting (e.g., XGBoost), and support vector machines, as well as deep learning (DL) methods including convolutional neural networks for imaging and natural language processing models, including large language models, for text data. Outputs include AI-assisted diagnosis of pneumonia and prediction of clinically relevant outcomes such as disease severity, respiratory failure, readmission, length of stay, and mortality

The objective of this review is to provide an examination focused on recent AI-based tools for pneumonia diagnosis and outcome prediction. We will discuss the various AI methodologies, including image-based techniques like CNNs and the use of predictive models for clinical decision support. Common input variables, prediction algorithms, and outcomes predicted by AI models are summarized in Fig. 1. This review aims to evaluate the current state of research, highlight key advancements, and identify challenges and opportunities for AI in pneumonia management.

## AI in Pneumonia Diagnosis

Utilizing AI has shown remarkable promise in diagnosing and identifying pneumonia. The diagnosis of pneumonia is often determined on a variety of factors, including clinical symptoms in addition to data from medical images, lab results, and vital signs.

### Pneumonia Diagnosis Using Clinical Data

Clinical data from the electronic health record (EHR) encompasses a wide range of patient information, including medical history, laboratory results, vital signs, and clinician notes, and is a growing source of data for ML models [15]. EHR-based AI tools typically analyze structured data (e.g., laboratory results, vital signs, medications) and unstructured data (e.g., clinician notes) to identify patterns indicative of pneumonia.

In a study conducted in the United Kingdom [16], classifications and regression trees (CART) as well as LR models were compared to predict community-acquired pneumonia (CAP) after respiratory tract infection using EHR data such as patient characteristics, medical management, antibiotic prescription, and laboratory tests, achieving an area under the receiver operating characteristic curve (AUROC) of 0.80 on a validation cohort. Heyman et al. [17] examined all dyspneic emergency department patients and developed a novel deep learning model, CareNet, to classify patients into heart failure, COPD exacerbation, and pneumonia, achieving AUROC of 0.87. Effah et al. [18] also utilized LR as well as seven other ML models to predict pneumonia based on demographic information, physical parameters (e.g., tachycardia, tracheal secretion, mean arterial pressure, etc.), and hematological parameters (e.g., c-reactive protein, procalcitonin, white blood cell count, etc.). Out of the eight models, RF and Extreme Gradient Boosting (XGBoost) had the highest accuracy with RF having an accuracy of 92.0% for the internal validation set and an accuracy of 88.6% for the external validation set. Dai et al. [19] compared ML algorithms of RF, LR, and Gradient Booster classifiers to differentiate COVID-19 from CAP based on clinical laboratory indicators with a high AUROC of 1.0 when using RF classifier or Gradient Booster classifier. Table 1 describes the studies in more detail.

**Table 1:** Recent published literature regarding utilizing models employing artificial intelligence for the diagnosis of pneumonia

STUDY	INPUT	SUBTYPES OF PNEUMONIA AND OTHER DIAGNOSES	ALGORITHMS	COMPARISON	STUDY DESIGN	BEST PERFORMANCE	SAMPLE SIZE	CALIBRATION	PERFORMANCE METRIC DETAILS
Sun et al. 2022 [16]	Clinical features such as age, BMI, Charlson Comorbidity Index	CAP	CART and LR	Read codes for pneumonia	Retrospective, UK CPRD GOLD database. No external validation	AUROC 0.81 [0.80, 0.84] for LR on test set and 0.80 [0.79, 0.81] on validation set	108,842 patients, 16,289 of whom had pneumonia	Hosmer-Lemeshow showing good calibration	Sensitivity and specificity not reported. Threshold details not reported.
Heyman et al. 2024 [17]	Clinical features such as medical history, prior diagnoses and comordities, smoking status, life management difficulties	Pneumonia (no subtypes specified), AHF, eCOPD, and other (all other diagnoses for dyspnea)	Clinical attention-based recurrent encoder network (CareNet)	ICD-10 codes	Retrospective, two EDs in the same region. No external validation	AUROC of 0.870 [0.848, 0.883] for diagnosis determination with one year of data	10,315 ED visits with 6,967 unique patients with 1,376 visits being classified as pneumonia	Calibration not reported. No DCA.	Sensitivity and specificity are reported. Threshold details not reported.
Effah et al. 2022 [18]	Clinical features such as age, symptoms, labs	Bacterial pneumonia	LR, SVM, AdaBoost, KNN, RF, XGBoost Naive Bayes, Multilayer Perceptron	Clinical diagnosis	Retrospective, single center. External validation performed with external dataset	AUROC 0.97 for XGBoost and 0.96 for RF on test set; 0.97 for XGBoost and 0.97 for RF on validation set.	535 patients	Calibration not reported. No DCA	Sensitivity and specificity not reported. Threshold details not reported.
*Rajpurkar et al. 2018 [21]	CXR	14 different lung pathologies including pneumonia (no subtypes specified)	CNN model with 121-layer DenseNet architecture	Physicians	Retrospective, ChestX-ray14 dataset [64]. External validation performed with external dataset	AUROC 0.851 [0.781, 0.911] for model. Outperformed radiologists; AUROC 0.823 [0.779, 0.856] for radiologists.	112,120 CXRs, with 420 radiologist-annotated validation images	Calibration not reported. No DCA	Implemented pathology-specific thresholds selected through maximization of the F1 score on the tuning set
*Rabbah et al. 2025 [22]	CXR	Pneumonia (no subtypes specified), COVID-19, and normal	CNN model with Inception V3 layer and Integrated Gradients method	Clinical diagnosis	Dataset collected from various sources. External validation performed with external dataset	AUROC of 0.960 on test set and 0.995 on validation set for model	5,856 images	Calibration not reported. No DCA	Sensitivity and specificity not reported. Threshold details not reported.
An et al. 2024 [32]	CXR	Pneumonia (no subtypes specified)	CNN model with attention ensemble using EfficientNet and DenseNet	Clinical Diagnosis	Retrospective, single center. No external validation	AUROC of 0.9564 for model on test set and 0.9314 +/- 0.0385 on validation set	5,856 images, of which 4,265 were classified as pneumonia	Calibration not reported. No DCA	Reports CI. Sensitivity and specificity reported. Threshold details not reported.
**Dominguez-Rodriguez et al. 2023 [23]	CXR	CAP and COVID-19	CNN Network	Physicians	Retrospective model development and prospective pilot test	AUROC of 0.79 for model	6,806 images	Calibration not reported. No DCA.	Sensitivity and specificity reported. Threshold details not reported.
Han et al. 2022 [35]	CXR	CAP and TB	Keras Deep Learning Toolkit and with parameters from Visual Geometry Group Network 16	Physicians	Retrospective, multicenter (3 different sites). No external validation performed	AUROC of 0.959 [0.943–0.976] for model	3,982 total cases, of which 1,488 in the CAP group	Calibration not reported. No DCA.	Reports CI. Sensitivity and specificity reported. Threshold details not reported.
Rixe et al. 2023 [34]	CXR	CAP	Word Embedding, SVM, XGBoost, LightGBM, Naive Bayes, LR	Radiologist interpretation and ICD-9 diagnosis code	Retrospective, multicenter (6 sites). No external validation	No AUROC reported. Accuracy of 93.6 for XGBoost when comparing to content experts	1,350 images	Calibration not reported. No DCA	Reports CI. Sensitivity and specificity reported. Threshold details not reported
Chapman et al. 2022 [72]	Combination of chest imaging radiographic features and EHR data including emergency department notes and discharge summaries	Pneumonia (no subtypes specified)	NLP model	Two physicians	Retrospective, multi center	No AUROC or accuracy reported. F1 score of 87.4 for the rule-based NLP system versus 69.8 for the transformer NLP model for the VA dataset. For the second and separate dataset, F1 score of 85.2 for the same NLP model and 89.8 for the modified NLP model that was tailored to this new dataset	800 documents from the VA system (200 emergency department notes, 400 radiology reports, 200 discharge summaries). For second dataset, 267 documents	Calibration not reported. No DCA	Reports sensitivity and specificity. CI not reported. Threshold details not reported
Panny et al. 2022 [69]	Combination of radiographic features (e.g. chest x-rays) and clinical data (medication history)	Pneumonia (no subtypes specified)	Model employing an NLP pipeline	Pulmonologists and ICD-9 / ICD-10 codes.	Retrospective, single database in integrated healthcare system comprised of 50 clinics and 7 hospitals. No external validation	No AUROC reported. Accuracy of 76.3%	91,998 episodes of pneumonia in 65,904 patients identified with a total of 225,893 chest x-ray reports	Calibration not applicable. No DCA	Sensitivity and specificity reported with p-values and CI reported
Yang et al. 2024 [36]	Chest CT in addition to clinical features	Severe CAP	Ada Boost, LR, RF, SVM, XGBoost, KNN, LightGBM, Naive Bayes	Physicians	Retrospective, single center. No external validation	AUROC of 0.8947 for Ada Boost when combining clinical features and radiologic features.	174 participants	Calibration not reported. No DCA	Sensitivity and specificity not reported. Threshold calculated at 0.7
Jiang et al. 2024 [20]	Chest CT	Severe CAP	LR, RF, KNN, SVM	Radiologists	Retrospective, single center. No external validation performed	AUROC of 0.975 for RF on test set and 0.857 on validation set.	115 patients	Calibration not reported. DCA showed favorable benefit at ranges 0.2–0.7 for the validation set	Threshold not reported. CI reported
*Wang et al. 2023 [37]	Chest CT	Bacterial, fungal, and viral pneumonia	HarDNet algorithm	Radiologists	Retrospective, multicenter (4 different sites). External validation performed with external dataset	AUROC of 0.961 for model on training set, 0.822 on test set, 0.877 on validation set Outperformed radiologists	2,763 participants	Calibration not reported. No DCA	Threshold not reported.
*Ouyang et al. 2020 [38]	Chest CT	CAP and COVID-19	Novel Online Attention Module with 3D ResNet34 model with a dual sampling strategy	RT-PCR	Retrospective, multicenter (8 sites). External validation performed for COVID-19	AUROC of 0.948 +/- 0.003 on test set, 0.988 on training set, 0.944 on COVID-19 validation set for model	3,645 patients, 4,982 images	Calibration not reported. No DCA	Reports CI. Threshold calculated at default of 0.5
Xie et al. 2023 [24]	Chest CT	Viral pneumonia, bacterial pneumonia, mycoplasma pneumonia, and COVID-19	CNN with three-level optimization framework	Standard clinical, radiological, culture/molecular assay results, and RT-PCR for COVID-19	Single dataset from 7 different sites. External validation performed	No AUROC reported. F1 score of 91.8% for model for detection of COVID-19 pneumonia and F1 score of 92.4% for detection of other types of pneumonia	2,218 images	Calibration not reported. No DCA	Sensitivity/specificity not reported. Threshold not reported
*Kessler et al. 2024 [25]	Ultrasound	Pneumonia (no subtypes specified)	CNN network with Visual Geometry Group-like network	Discharge diagnosis with imaging (CXR and/or CT)	Prospective, multicenter (2 sites). No external validation	No AUROC reported. Accuracy of 88.5% for model	107 participants, 117 exams, 604 positive videos, 589 negative videos	Calibration not reported. No DCA	Sensitivity and specificity reported. Threshold not reported
Meng et al. 2025 [46]	Plasma metabolic fingerprints enhanced by platinum copper alloys	Severe CAP	Lasso Regression, Ridge Regression, and Neural Networks	Clinical diagnosis	Retrospective, multicenter (3 sites). External validation performed with external dataset	AUROC of 0.832 for model; when biomarkers were implemented, AUROC increased to 0.846.	69 individuals	Calibration not reported. No DCA	Sensitivity and specificity reported. Threshold not reported

Abbreviations: *BMI* body mass index, *CAP* community acquired pneumonia, *UK* United Kingdom, *CPRD* Clinical Practice Datalink, *AUROC* area under the receiver operating characteristic curve, *CI* confidence interval, *AHF* acute heart failure, *eCOPD* exacerbation of chronic obstructive pulmonary disease, *ICD* International Classification of Diseases, *ED* emergency department, *DCA* decision curve analysis, *CXR* chest x-ray, *CT* computed tomography, *RT-PCR* reverse transcriptase polymerase chain reaction. For models mentioned: *CART* Classification and Regression Trees, *LR* Logistic Regression, *RF* Random Forest, *XGBoost* Extreme Gradient Boosting, *SVM* Support Vector Machine, *AdaBoost* Adaptive Boosting, *KNN* K-Nearest Neighbors, *CNN* Convolutional Neural Network, *NLP* Natural Language Processing * of importance; ** of major importance

No single modeling approach consistently outperformed others across all clinical contexts, outcomes, and datasets; each study noted different algorithms with the highest AUROC. This highlights that the performance of individual algorithms are context dependent and can vary depending on factors such as sample size, feature dimensionality, outcome prevalence, and validation strategy. Some studies have noted strong performance with ensemble methods such as XGBoost or RF [18, 20] while others have demonstrated comparable discrimination using LR [16] or neural network-based approaches [21–25].

Furthermore, across the included literature on pneumonia diagnosis, the most frequently reported top predictor included procalcitonin. This finding is not surprising, as procalcitonin has been utilized in diagnostic discrimination between viral and bacterial pneumonia [26] though guidelines caution against its sole use as a diagnostic tool but can aid in antibiotic de-escalation [27]. Studies also identified C-reactive protein, albumin to globulin ratio, uric acid, white blood cell count, neutrophil count, basophil count, red blood cell count, and mean corpuscular hemoglobin concentration as secondary but clinically relevant contributors to model performance. While the relative ranking and effect magnitude varied depending on cohort characteristics, outcome definitions, and modeling approaches, these features were repeatedly associated with improved discrimination and calibration.

One of the main advantages of using EHR data for pneumonia diagnosis is the ability to continuously update and refine AI models with real-time patient data. As more information becomes available over time, these models can learn and adapt, offering more accurate predictions for individual patients. However, challenges remain, such as ensuring the quality and completeness of EHR data and addressing issues like data fragmentation or inconsistency between different healthcare systems [28]. Despite these challenges, EHR-based AI tools hold significant potential to complement imaging techniques, improving both the speed and accuracy of pneumonia diagnosis in clinical settings.

### Pneumonia Diagnosis Using Imaging Data

In addition to using EHR data for diagnosis of pneumonia, using ML and DL models to make a diagnosis based on different imaging modalities has been an impressive area of research. Radiomics models have also been increasingly used in imaging-based diagnoses. Radiomics was a concept proposed by Lambin et al. in 2012 [29] where feature information can be extracted from medical images; this data can then be used to train models for specific tasks. Some prominent radiomics features cited by studies have included shape based features, gray-level dependence matrix features, gray-level size zone matrix features, and gray-tone difference matrix features. CNNs are often also used in imaging-based tasks including medical images, which have important applications for radiology [30]. CNNs are particularly effective for tasks like image classification, where they can automatically identify patterns and features within images without the need for manual feature extraction or human supervision [31]. In the context of pneumonia diagnosis, CNNs are trained on large datasets of annotated medical images, such as chest X-rays (CXRs) or CT scans, to recognize specific characteristics associated with pneumonia, such as lung consolidation, infiltrates, or pleural effusion. CNNs operate by passing an image through multiple layers, each responsible for extracting increasingly complex features from the raw image data. The initial layers may detect simple edges or textures, while deeper layers identify more intricate patterns, such as the shape of lung abnormalities.

### Chest X-ray-based Diagnosis

For diagnosis from CXRs, one of the prominent DL models that yielded an accuracy similar to that of radiologists was CheXneXt [21]. This CNN model was able to detect the presence of multiple different lung pathologies, including pneumonia with a performance comparable to radiologists (Model AUROC was 0.851 compared to radiologist AUROC of 0.823). This study was pivotal in proving the utility of CNNs on CXRs and has influenced other research. For example, a recent 2025 study [22] cites one of the drawbacks of DL models like CheXneXt is the lack of transparency and interpretability, and generates a DL model to address these weaknesses. The DL model used Integrated Gradients in order to provide more interpretability of the image, which yielded as high of an accuracy as 97.2%. Integrated Gradient is a technique that assesses the importance of each individual region within an image towards the final model classification. Many recent studies have been focusing on using novel methods to improve the performance of ML models. Another study [32] focused on improving a CNN model uses an attention ensemble and capitalizes on the strength of two different mechanisms, EfficientNet and DenseNet, which was able to yield an accuracy of 95.2%. Indeed, a recent meta-analysis of 15 studies published in 2025 has demonstrated the promising capabilities of AI algorithms in pneumonia detection; the pooled sensitivity and specificity for pneumonia diagnosis reported was 88% and 90%, respectively [33].

Some studies have focused on Natural Language Processing models (NLPs) of radiology reports; a 2023 study [34] analyzed the accuracy of six NLP models, including word embedding, SVM, XGBoost, light gradient boosting, Naive Bayes, and LR, with Naive Bayes demonstrating the highest sensitivity of 93.5%. Other AI models have not only been able to diagnose pneumonia based on CXR but also differentiate pneumonia from other lung conditions. Han et al. [35] reported a CNN-based model to distinguish active pulmonary tuberculosis, CAP, and healthy lungs. Other studies have examined the applicability and usability of the model in real life. Dominguez-Rodriguez et al. [23] assessed a CNN model in comparison to pediatricians reviewing CXRs and found a sensitivity of 90.9% for the model. This study then analyzed three medical residents using a support decision tool based on the CNN model, which found a higher agreement between the medical residents when using the tool.

### CT-Based Diagnosis

While there has been a focus on CXRs, AI models have also demonstrated good performance for diagnosing pneumonia using CT scans. Yang et al. [36] and Jiang et al. [20] utilize a radiomics-based model to extract different features from CT scans in order to make the diagnosis of severe CAP, achieving high performance with AUROCs in the 0.85 range. A novel DL algorithm called PneumoniaPlus was proposed [37], which was among the first models to distinguish between viral, bacterial, and fungal pneumonia, and boasted a performance comparable to radiologists. Furthermore, in 2020 during the height of the COVID-19 pandemic, Ouyang et al. [38] demonstrated the capabilities of a dual-sampling attention network, which relies on training two separate 3D ResNet34 mechanisms and integrating predictions from both mechanisms to differentiate COVID-19 from CAP using CT scans, yielding a AUROC value of 0.94. As an attempt to further improve ML model performance, a 2023 study [24] employed a three-level optimization method. This model yielded an F1-score of 91.8% in detecting COVID-19 pneumonia and F1-score of 92.4% for detection of other types of pneumonia, which was able to outperform 6 other previous models [39,40,41,42,43,44].

### Ultrasound-based Diagnosis

Though CT scans and CXRs have been more established areas of AI models for pneumonia diagnosis, a developing area of research in AI and imaging-based diagnosis is the use of ultrasound imaging for pneumonia. Kessler et al. [25] used a CNN-network to detect consolidation on ultrasound to diagnose pneumonia. Using ultrasound video yielded a sensitivity of 88% and specificity of 89% for detecting consolidation.

Despite significant advances, AI models still face challenges in differentiating pneumonia from these other conditions. Variability in image quality, the presence of comorbidities, and different stages of disease can impact the accuracy of AI predictions. Furthermore, while AI can recognize patterns on images, it still relies on comprehensive datasets and external clinical data to make a definitive diagnosis. Though no current ML models have been approved for FDA use in the diagnosis of pneumonia using clinical data, there are some for imaging modalities such as the Exo Iris ultrasound to detect lung consolidation [45].The use of AI has been rapidly improving, and over time, the implementation of these models may become more widespread, and novel methods will be further explored. A recent 2024 study [46] examined using ML models and porous platinum-copper alloys to extract plasma metabolic fingerprints to diagnose severe CAP. The intersection of ML models and the diagnosis of pneumonia will continue to be an exciting and emerging area of research.

## AI to Predict Patient Outcomes

AI has also shown promise in predicting patient outcomes in pneumonia, which is crucial for optimizing treatment strategies, resource allocation, and improving patient care. There have been increasing recent efforts in applying AI to pneumonia outcome prediction, aiming to capture complex interactions of clinical features and achieving better accuracy at outcome prediction that potentially outperforms existing scoring systems. By analyzing a variety of data inputs, AI models can help clinicians predict disease severity, the likelihood of mortality, and other important outcomes like hospital length of stay or readmission risk. These predictions not only enhance clinical decision-making but also aid in developing personalized treatment plans for patients with pneumonia. Major articles published since 2020 that implement AI models for pneumonia outcome prediction have been summarized in Table 2.

**Table 2:** Major articles published since 2020 that implement AI models for pneumonia outcome

STUDY	INPUT VARIABLES	SUBTYPES OF PNEUMONIA	ALGORITHMS	COMPARISON WITH EXISTING PREDICTION SYSTEM	STUDY DESIGN	OUTCOMES STUDIED	BEST PERFORMANCE	SAMPLE SIZE	KEY FINDINGS	CALIBRATION
*Jones et al. 2021[47]	Various subsets of PSI variables	CAP	LR, spline, XGBoost	PSI score without information on mental status or radiograph	Retrospective cohort (VA multi-center EHR, 117 sites)	30-day mortality	AUROC CI [0.86–0.87]	297,498 encounters	XGBoost outperforms traditional methods	Good for all models. ML is better at avoiding underestimating death in low-risk quantiles
Kang et al. 2020 [48]	CURB-65 variables; additional demographics, vital signs, labs, radiology, comorbidities for extensive models	All types	RF	CURB-65	Retrospective cohort, single center (ED registry)	30-day mortality, ICU admission	AUROC 0.844 [0.843–0.845]	1974 patients	RF outperforms CURB-65; RF with extensive variables outperforms RF using only CURB-65 variables	Not reported
Odeyemi et al. 2024 [49]	Demographics, vital signs, labs, comorbidities, PSI, SOFA, APACHE III variables (within first 6 h)	CAP	GBM	PSI, CURB-65	Retrospective cohort, single center	Need for advanced respiratory support, in-hospital mortality	AUROC 0.713; accuracy 61.2% [59.5%–62.8%]	4379 patients	GBM outperforms PSI and CURB-65	Not reported
Lyu et al. 2023 [50]	Demographics, vital signs, lab, comorbidities, APACHE III (within first 1 h)	All types	LightGBM, XGBoost, CatBoost, RF	None	Retrospective, database (eICU-CRD)	Early respiratory failure after ICU admission	AUROC 0.858 ± 0.050	1676 patients	CatBoost outperforms other ML models	Not reported
Wang et al. 2022 [51]	PSI variables	CAP	Decision Trees, RF, Gaussian Naive Bayes, LR, Linear Discriminant Analysis, Stochastic Gradient Descent, SVM, KNN, and Multi-Layer Perceptron	PSI	Retrospective, multi-center (749 US hospitals)	30-day mortality	AUROC 0.82 [0.81, 0.84]	34,720 patients	RF outperforms PSI	Not reported
Lee et al. 2025 [76]	Demographics, clinical diagnoses, labs, medications, procedures, hospital visit types and frequencies	CAP	LASSO LR, GBM, RF, AdaBoost, and stacked ensemble model	None	Retrospective, feature learning (CDM), hospitalization data	All-cause in-hospital mortality	AUROC 0.867 [0.823–0.910]	2594 patients	The stacked ensemble model achieves the best prediction performance	Not reported
Xu et al. 2022 [52]	Demographics, vital signs, labs, comorbidities	CAP	Decision Trees, RF, XGBoost, SVM, Naive Bayes, KNN, Ridge regression, LR, NN	None	Secondary analysis on prospective registry cohort (three cities, South America)	Hospital admission, mortality, ICU admission, 1-year post-enrollment status	AUROC 0.801 to predict ICU admission; AUROC 0.831 to predict death	2302 patients	XGBoost is best performing for ICU admission. NN is best performing for death.	Not reported
Zhao et al. 2025 [53]	Demographics, vital signs, labs (first 24 h), Medical history, comorbidities, all APACHE-II, CURB-65, PSI and SOFA variables.	Severe pneumonia	LightGBM, SVC, RF, LR	APACHE-II, CURB-65, PSI and SOFA	Retrospective, single-center	In-hospital all-cause mortality	AUROC 0.8779 [0.738 to 0.974]	875 patients	Ensemble model outperforms APACHE-II, CURB-65, PSI, and SOFA	The ensemble model is well calibrated for predicting death.
Feng et al. 2021 [54]	62 clinical feature variables: demographics, clinical diagnostic features, treatment features	CAP	FCNN; LR, SVM, KNN, Gaussian naive Bayes, decision trees, RF, and their integrated stacking patterns	None	Retrospective, single-center	In-hospital mortality	AUROC 0.975 (confidence interval not reported)	3997 patients	Ensemble FCNN model outperforms other algorithms	Not reported
Li et al. 2024 [77]	Demographics, vital signs, labs, and other clinical variables manually selected based on LASSO regression method	All types	XGBoost	APACHE	Retrospective, eICU-CRD database	In-hospital mortality	AUROC 0.778 ± 0.016	10,962 patients	XGBoost performed better than traditional scoring systems	Not reported
Aldhoayan et al. 2022 [78]	The history of pneumonia admissions, vital signs, radiology, laboratory, medication list, and comorbidities	CAP	RF, decision trees, SVM, LR	None	Retrospective observational, single healthcare organization	30-day readmission	Accuracy of 90%	5,776 patients	Multiple models achieved accuracy of 90%	Not reported
Jeon al. 2023 [55]	Demographics, BMI, smoking history, comorbidities, vital signs, laboratory, prognostic scores, and treatment with antibiotics or steroids	Severe pneumonia	LR, LightGBM, Multilayer Perceptron	SOFA, SAPS II, and APACHE II scores	Retrospective, single-center	In-ICU mortality	AUROC 0.838 [0.791–0.884]	816 patients	Their multilayer perceptron model outperformed SAPS II	Reported in Supplement only, raw Brier scores 0.142–0.170
*Pan et al. 2025 [56]	Demographics, comorbidities, APACHE II, SOFA, laboratory, other clinical features (worst values within first 48 h)	Severe CAP	LR, RF, XGBoost, LightGBM, and SVM	APACHE II	Retrospective, two-center (development + external validation)	In-hospital mortality	AUROC 0.842 (95% CI: 0.757–0.927)	Development: 455, external validation: 120	Their LightGBM model performed better than APACHE II	Not reported
Yuan et al. 2022 [79]	Demographics, comorbidities, vitals, laboratory	CAP	RF, XGBoost, Broad Learning System (BLS), Deep Neural Networks, CNN	None	Retrospective, single-center case-control	28-day mortality	AUROC 0.962 (95% CI: 0.936–0.988)	1,210 patients	Their broad learning system model outperformed other methods	Not reported
Sheu et al. 2022 [62]	Demographics, vitals, laboratory, chest X-ray images	CAP	GBM, L2 LR, RF, SVM, convolutional neural filters, CNN, autoencoders	None	Not completely specified, but hospital-based	Early (< 7d) vs. late (> 7d) discharge (proxy: length of stay)	Accuracy of 0.92 using the CXR model, AUROC of SVM model 0.64–0.68	3972 patients	Their model has potential to optimize patient discharge	Plots provided showing reasonable calibration
Kim et al. 2023 [59]	CXR images	CAP	CNN	CURB-65	Retrospective, multi-center	30-day mortality	AUROC 0.83 (95% CI, 0.80–0.87)	Development: 7105 patients; temporal validation: 947, external validation 467, 381	Their model outperformed CURB-65 in the temporal test set	Plots demonstrate undercalibration in external test sets
Li et al. 2023 [57]	Demographics, vitals, comorbidities, labs, pathogen, treatment, and clinical scores	Severe CAP, age > 65	Stepwise regression	SOFA, CURB-65, SOAR, PSI	MIMIC-III	30-day In-hospital mortality	AUROC 0.751 (95% CI 0.749–0.752)	619 patients	Their model performed SOFA, SOAR, CURB-65	Not reported
Shin et al. 2024 [58]	CXR AI-consolidation score, CURB-65, PSI, initial O2 requirement, intubation	All types	Multivariate Cox regression	CURB-65, PSI	Retrospective, single site	30-day mortality	AUROC 0.726 (95% CI 0.644–0.809)	489 patients	Their model outperformed CURB-65 and PSI	Visualization provided, integrated Brier score 0.088
Cilloniz et al. 2023 [60]	Demographic variables, comorbidities, and physiologic parameters	CAP	Causal probabilistic network	PSI, SOFA, qSOFA, CURB-65	Retrospective, multi-site	30-day mortality	AUROC 0.826	Derivation: 4,531 patients; validation: 1,034	Their algorithm, SeF-ML, outperformed CURB-65 and qSOFA but not PSI or SOFA	Poorly calibrated by Hosmer-Lemeshow but visually appears well-calibrated

Abbreviations: *CAP* community-acquired pneumonia, *VAP* ventilator-associated pneumonia, *HAP* hospital-acquired pneumonia, *AUROC* Area Under the Receiver Operating Characteristic Curve, *CURB-65* confusion, urea, respiratory rate, blood pressure, age ≥ 65, *PSI* pneumonia severity index, *SOFA* sequential organ failure assessment, *LR* logistic regression, *XGBoost* extreme gradient boosting, *GBM* gradient boosting machine, *RF* random forest, *SVM* support vector machine, *KNN* K-Nearest Neighbors, *LASSO* Least Absolute Shrinkage and Selection Operator, *AdaBoost* adaptive boosting, *NN* neural network, *FCNN* fully connected neural network, *eICU-CRD* eICU Collaborative Research Database, and *MIMIC* Medical Information Mart for Intensive Care. * of importance; ** of major importance

Across studies, tree-based methods such as gradient boosting and random forest are the most commonly implemented algorithms for pneumonia outcome prediction [47–51]. These approaches tend to outperform traditional statistical models such as logistic regression, likely because they can capture nonlinear relationships and complex feature interactions within high-dimensional clinical datasets. Across published studies, tree-based models frequently achieve AUROC values in the range of approximately 0.80–0.90 for mortality prediction [50–56], whereas logistic regression models demonstrated comparable or slightly lower performance. Neural network architectures have also been explored, particularly when incorporating imaging data, although their performance advantage over ensemble tree methods has been inconsistent. As a result, gradient boosting and random forest remain the most widely adopted approaches in current pneumonia outcome prediction models.

Several studies have also examined model interpretability using feature importance ranking or SHAP (Shapley Additive Explanations) analysis to identify the most influential predictors of outcomes. Across studies, commonly identified predictors include older age, markers of physiologic instability such as temperature, respiratory rate, and systolic blood pressure, renal function markers such as blood urea nitrogen and creatinine, laboratory markers of physical stress such as lactic acid and pH, and comorbid conditions including chronic lung disease and cardiovascular disease [49, 50, 52–54]. Feng et al. [54] included antibiotic types in their models, and were important features based on their feature weight analysis. These findings are broadly consistent with established pneumonia severity scoring systems, suggesting that AI models often identify clinically plausible predictors while leveraging additional interactions between variables to improve predictive performance.

### Mortality Prediction

Mortality is the most common outcome measure that AI algorithms attempt to predict. ML models have been developed to predict both short-term (e.g., 30-day mortality) and in-hospital mortality. The vast majority of studies focus on CAP, likely because its early clinical features are more homogeneous and established risk stratification tools are more readily available for benchmarking model performance. Common clinical features included for model building are demographics (age, sex), comorbidities, vital signs, lab results, and treatment. Different studies select for clinical features of interest in different ways. Some select for input variables based on preliminary analyses such as logistic regression [57], while some others refer to variables that contribute to existing clinical scoring systems such as PSI, APACHE III, etc. [47, 49]. There are a few studies that also included CXR findings in mortality prediction models [58, 59]. Most implemented algorithms include gradient boosting and random forest, and some studies assess performance of ensemble models. AI models achieve variable AUROC ranging from 0.69 to 0.92 [52, 59]. They can offer clinicians a predictive estimate of mortality risk early in the course of the disease, which can guide treatment decisions such as the escalation of care, use of life-sustaining interventions, or even discussions regarding palliative care. AI models can be integrated with established clinical scoring systems, such as CURB-65 or PSI, to improve predictive accuracy. These scoring systems are widely used to assess the risk of mortality, but AI models can enhance their utility by incorporating more complex data patterns and providing real-time, individualized predictions. Many papers compared their results directly against CURB-65 and PSI, with a range in improvement over these scores of 0.04–0.40 [47–50, 57–60], though noting the inherent bias in papers likely only being published or reporting results that outperformed these classical scoring systems.

### Pneumonia Severity Prediction

One of the key outcomes that AI models aim to predict is the severity of pneumonia, most commonly characterized by hospital admission, ICU admission, respiratory failure, need for advanced respiratory support such as high-flow supplemental oxygen, noninvasive ventilation, and mechanical ventilation. AI-based prediction models can integrate a variety of input features to predict the likelihood of these severe outcomes [48–50, 52]. Kang et al. [48] used EHR data and radiographic images collected from the emergency department to predict the composite outcome of ICU admission and 30-day mortality among patients with CAP. Their random forest models showed promise at predicting this composite outcome indicative of severe CAP, achieving AUROC as high as 0.84 compared with 0.62 using CURB-65. Odeyemi et al. [49] examined CAP patients who were already hospitalized and used EHR data from the first six hours of hospitalization to predict the need for advanced respiratory support or mortality. Their gradient-boosted machine achieved an AUROC of 0.71, superior to that of PSI (0.65) and CURB-65 (0.62). More recently, efforts using large language models (LLMs) are beginning to emerge, with promising results from including radiology reports [61]. These studies show the potential of AI models to outperform existing predictive tools with more complex early clinical data in predicting the severity of pneumonia.

### Length of Stay (LOS) and Readmission Prediction

In addition to predicting severity and mortality, some studies have started to use AI models to predict LOS and readmission. Predicting the LOS is essential for managing hospital resources, as it can help in discharge planning and capacity management, particularly in high-demand settings like intensive care units. Sheu et al. [62] implemented a multi-model data analysis for pneumonia status prediction, where the model sequentially checked patients’ vital signs and CXR to assess pneumonia status on a certain day. With this approach, this study achieved an accuracy of 0.75 to predict early discharge (< 7 days) vs. late discharge (> 7 days). Similarly, AI models trained on historical patient data can predict the likelihood of hospital readmissions following a pneumonia episode. Huang et al. [63] used an SVM trained on EHR data to predict 30-day all-cause readmission for patients hospitalized with pneumonia. By analyzing age, gender, number of medications, length of admission, number of comorbidities, and total admission cost, this model achieved an accuracy of 83.95% in predicting readmission. There are a few other studies that attempt to use AI methods to predict 30-day readmission among pneumonia patients, but the overall accuracy of prediction was modest [64]. Readmission risk is typically associated with factors such as incomplete treatment, early discharge, or the presence of comorbid conditions that complicate recovery. By predicting readmission risk, AI can guide clinicians in providing tailored discharge plans and post-discharge follow-up, reducing the chances of readmission and improving long-term outcomes for patients.

Overall, by providing clinicians with timely, data-driven insights into disease severity, mortality risk, and other important outcomes, AI can enhance the decision-making process and ensure that resources are allocated efficiently. As AI continues to evolve, its ability to integrate diverse data sources and provide personalized predictions will be an invaluable tool in improving the care and management of patients with pneumonia.

## Key Challenges and Limitations

While the potential of AI in pneumonia diagnosis and outcome prediction is immense, several challenges and limitations must be addressed to ensure the safe, effective, and equitable implementation of these technologies in clinical settings. Key challenges include issues related to data quality and bias, model generalizability, interpretability and explainability, and regulatory and ethical considerations.

Several studies reported exceptionally high AUROC values, in some cases approaching near perfect performance, for example, Effah et al. [18] reported AUROC 0.97 for XGBoost, Rabbah et al. [22] reported 0.995 on validation, Feng et al. [54] reported 0.975. Such results should be interpreted with caution, as they may reflect methodological issues such as overfitting, data leakage, small and non-representative test sets. Several studies had small sample sizes, for example, Meng et al. [46] (*n* = 69), Kessler et al. [25] (*n* = 107), and Yang et al. [36] (*n* = 174).

There was a paucity of studies that provided key metrics such as calibration and decision curve analysis, or even confidence intervals, only a few such as Sun et al. [16], Zhao et al. [53] and Kim et al. [59] reported clear calibration and only Jiang et al. [20] reported clear DCA analysis. Furthermore, a large majority of the studies discussed were retrospective studies with very few prospective studies having been conducted; Kessler et al. [25] is the clearest prospective example by enrolling pediatric patients with suspicion of lower respiratory tract infection and Dominguez-Rodriguez et al. [23] included a pilot test prospectively conducted. While retrospective studies can provide important insight, prospective studies can be valuable for mitigating potential biases and confounding factors. Other key factors when assessing these different models include the presence of external validation, which can often verify the reproducibility of the results. Most studies were single center [32, 36, 48, 55], with Jones et al. [47] standing out by including 117 sites across the Veterans Affairs Healthcare system.

One of the primary challenges in developing AI models for pneumonia diagnosis and outcome prediction is ensuring the quality and representativeness of the data used to train these models [65]. If the training data is of poor quality or contains errors, the model’s performance will be compromised. For example, incomplete or inconsistent medical records, missing data on patient history, or incorrect lab values can lead to inaccurate predictions [66]. Many studies had limited information on data collection processes and data provenance, raising concerns about reproducibility and biases. Moreover, bias in the data can significantly impact the model’s outcomes, particularly if certain populations (e.g., racial or ethnic minorities, rural populations, or underrepresented age groups) are underrepresented in the training datasets [67]. This can result in AI models that perform poorly for these groups, exacerbating existing healthcare disparities and leading to inequitable healthcare delivery.

Another fundamental challenge in developing AI models for pneumonia diagnosis and outcome prediction is the inconsistency and vagueness in how pneumonia is labeled and classified. This is highly heterogeneous even amongst randomized controlled trials’ diagnostic criteria [68]. Pneumonia can present with a wide range of symptoms, severity levels, and radiological findings, making its diagnosis subjective and sometimes inconsistent across healthcare providers. Clinicians may use different criteria to diagnose pneumonia, and there is no universal standard for defining the disease, which can lead to variability in labeling, especially in mild cases or early-stage pneumonia [68]. Many studies relied on administrative codes [17, 34, 69], which have been known to suffer from miscoding and variable sensitivity. This inconsistency in labeling poses a significant challenge for training AI models, as they require clear and standardized labels for accurate learning.

Currently, the vast majority of published studies in pneumonia diagnosis and outcome prediction are single-center retrospective studies without good external validation, and even fewer model deployments [70]. Models trained in one hospital or healthcare system may not perform as well when deployed in another institution with different patient populations, care protocols, or data systems. Factors such as variation in imaging techniques, laboratory test methods, and clinical practices can all influence the model’s ability to make accurate predictions in new settings. Additionally, discrepancies in EHR systems and data formats can hinder the model’s ability to integrate and process data effectively. To address this challenge, AI models must be rigorously validated across multiple institutions and patient demographics, ensuring that they can generalize well and provide consistent, reliable results regardless of where they are applied.

The literature in adult pneumonia NLP remains dominated by radiology report–based diagnosis [71] and case identification [72, 73] with limited work for surveillance, and early detection uses. Outcome prediction from unstructured notes remains limited as well, though there have been some preliminary efforts to predict outcomes using admission textual data [74] or pair it with structured data using ensemble models to predict outcomes [75]. Given the emergence of clinical applications of large language models, this is an exciting area for future work.

The integration of AI into healthcare raises several regulatory and ethical concerns that must be carefully considered. Regulatory frameworks for the approval and use of AI in medicine are still evolving, and many countries lack standardized guidelines to ensure the safety, efficacy, and privacy of AI-based tools. In particular, the use of patient data for training AI models must comply with stringent privacy regulations, such as the Health Insurance Portability and Accountability Act (HIPAA) in the United States or the General Data Protection Regulation (GDPR) in Europe. Additionally, AI models must undergo rigorous clinical validation to ensure they are safe and effective before they are used in real-world clinical practice. Ethical concerns also arise in relation to the potential for AI to replace human decision-making, leading to a reduced role for clinicians or contributing to overreliance on technology. It is essential that AI is viewed as a complementary tool, not a replacement for human judgment, and that its use does not compromise the doctor-patient relationship or decision-making autonomy.

Despite promising model performance, successful clinical translation requires careful consideration of regulatory, technical, and human factors. AI tools for pneumonia diagnosis and prognosis would likely fall under the FDA Software as a Medical Device, necessitating rigorous validation, documentation, and ongoing post-deployment monitoring. In practice, these models could be integrated into clinical workflows as real-time alerts, decision support tools, or screening systems embedded within EHR. However, implementation remains challenging due to barriers such as interoperability with EHR systems, the need for clinician trust and interpretability, and the risk of alert fatigue if models are not thoughtfully designed and validated in real-world settings. Overall, the majority of models around pneumonia remain in preliminary development stages and have not been subjected to prospective or true deployment testing, highlighting a substantial gap between model development and real-world application.

While AI offers tremendous promise in the diagnosis and outcome prediction of pneumonia, these challenges and limitations must be addressed to ensure its successful integration into healthcare. Overcoming data quality and bias issues, improving model generalizability, ensuring interpretability, and addressing regulatory and ethical concerns are essential steps toward achieving the widespread, equitable, and safe deployment of AI in clinical practice. By focusing on these challenges, researchers, clinicians, and policymakers can work together to realize the full potential of AI in improving patient care and outcomes.

## Conclusion

AI is increasingly transforming the landscape of pneumonia diagnosis and prognosis, offering rapid, accurate, and data-driven insights that enhance clinical decision-making. This review has highlighted the breadth of AI applications—from image-based diagnostic tools leveraging convolutional neural networks to multimodal prognostic models that integrate EHRs, laboratory data, and notes. There are many exciting prospects regarding the use of AI in the diagnosis and prognosis of pneumonia. Many ML models have proven to show high precision and accuracy regarding the diagnosis of pneumonia utilizing imaging and clinical data, and the combination of both has yielded even greater accuracy. Further research will be needed for eventual formal validation of these models and for everyday use in the clinical setting. Some have already attempted to assess the real world applicability [23], and future studies will need to continue to examine different models’ usability in real life, especially models with integration of multimodal data.

## Key References

- Domínguez-Rodríguez S, Liz-López H, Panizo-LLedot A, et al (2023) Testing the performance, adequacy, and applicability of an artificial intelligence model for pediatric pneumonia diagnosis. Comput Methods Programs Biomed 242:107765
  - ○ This CNN-based model for distinguishing CAP from COVID-19 included over 6,000 chest radiographs and included a prospective pilot test. Beyond reporting an AUROC of 0.79, the authors uniquely assessed real-world applicability by evaluating whether medical residents using the model as a decision support tool achieved better inter-rater agreement than residents working without it.
- Kessler D, Zhu M, Gregory CR, et al (2024) Development and testing of a deep learning algorithm to detect lung consolidation among children with pneumonia using hand-held ultrasound. PLoS One 19:e0309109
  - ○ This is one of the few prospective, multicenter pneumonia AI studies in the reviewed literature. The authors developed a CNN with a Visual Geometry Group-like architecture to detect consolidation on point-of-care ultrasound in pediatric patients, using discharge diagnosis with imaging confirmation as the reference standard. With 117 exams generating 604 positive and 589 negative videos, the model achieved 88.5% accuracy. The work expands AI-based pneumonia diagnosis beyond CXR and CT into a portable, radiation-free imaging modality with particular relevance for low-resource and pediatric settings.
- Pan J, Guo T, Kong H, Bu W, Shao M, Geng Z (2025) Prediction of mortality risk in patients with severe community-acquired pneumonia in the intensive care unit using machine learning. Scientific Reports 15:1–15
  - ○ This two-center study compared LR, RF, XGBoost, LightGBM, and SVM for in-hospital mortality prediction in severe CAP, using APACHE II as the comparator. The LightGBM model achieved AUROC 0.842 (95% CI 0.757–0.927) and outperformed APACHE II. The study is notable for performing external validation (development cohort n=455, external validation n=120) and for releasing a web-based application of the model—a step toward clinical implementation that few of the reviewed prognostic studies have taken.
- Rabbah J, Ridouani M, Hassouni L. Improving pneumonia diagnosis with high-accuracy CNN-Based chest X-ray image classification and integrated gradient. Biomed Signal Process Control. 2025;101:107239
  - ○ Using a CNN built on the Inception V3 architecture and trained on 5,856 images to distinguish pneumonia, COVID-19, and normal chest radiographs, this study achieved AUROC 0.96 on the test set and 0.99 on validation. Beyond performance, the work is notable for explicitly addressing the interpretability gap in deep learning by integrating the Integrated Gradients attribution method, which highlights image regions driving predictions.
- Essa ME. Diagnostic accuracy of AI in chest radiography for pneumonia and lung cancer: A meta-analysis. Eur J Radiol Open. 2025;15:100701.
  - ○ This meta-analysis of 15 studies and approximately 12,000 chest radiographs provides pooled estimates of AI diagnostic performance for pneumonia detection (sensitivity 88%, specificity 90%). It offers a quantitative synthesis that complements the narrative review by establishing population-level performance benchmarks against which individual studies can be compared.

## Data Availability

No datasets were generated or analysed during the current study.

## Declarations

### LLMs

ChatGPT and Claude were used to assist with outlining, drafting, reviewing, and rewording. Undermind.ai was used to help in literature search and review. The authors reviewed and edited all content and take responsibility for final content.
